# Programmable Deployment of Tensegrity Structures by Stimulus-Responsive Polymers

**DOI:** 10.1038/s41598-017-03412-6

**Published:** 2017-06-14

**Authors:** Ke Liu, Jiangtao Wu, Glaucio H. Paulino, H. Jerry Qi

**Affiliations:** 1 School of Civil and Environmental Engineering, Georgia Institute of Technology 5142B Jesse W. Mason Building, 790 Atlantic Drive NW, Atlanta, GA 30332 USA; 2 George W. Woodruff School of Mechanical Engineering, Georgia Institute of Technology 801 Ferst Drive MRDC 4104, Atlanta, GA 30332 USA

## Abstract

Tensegrity structures with detached struts are naturally suitable for deployable applications, both in terrestrial and outer-space structures, as well as morphing devices. Composed of discontinuous struts and continuous cables, such systems are only structurally stable when self-stress is induced; otherwise, they lose the original geometrical configuration (while keeping the topology) and thus can be tightly packed. We exploit this feature by using stimulus responsive polymers to introduce a paradigm for creating actively deployable 3D structures with complex shapes. The shape-change of 3D printed smart materials adds an active dimension to the configurational space of some structural components. Then we achieve dramatic global volume expansion by amplifying component-wise deformations to global configurational change via the inherent deployability of tensegrity. Through modular design, we can generate active tensegrities that are relatively stiff yet resilient with various complexities. Such unique properties enable structural systems that can achieve gigantic shape change, making them ideal as a platform for super light-weight structures, shape-changing soft robots, morphing antenna and RF devices, and biomedical devices.

## Introduction

Deployable structures have important applications, such as space structures^1–3^, robotics^4, 5^, morphing antenna and RF devices^6^, and biomedical devices^7^. Integrated only by self-stress, tensegrity^8, 9^ structures are inherently deployable^1, 10, 11^. They do not require mechanisms to lock the deployed shape, as many other deployable systems do, because the self-stresses also provide structural stability^12, 13^. As the struts are connected by flexible cables, complex articulated joints that are typical in truss-made or origami-inspired deployable structures are also circumvented. These features apply to both terrestrial^11, 14^ and outer-space structures^1, 15^, scaling from nanometers^16^ to meters^2^. Beyond deployability, tensegrity displays aesthetic formation^8^, high-precision controllability and easy tunability^11, 14^. In nature, tensegrity structures are found in living systems and play an important role to the fundamental structure and function of cells^17, 18^.

Recently, advanced additive manufacturing technologies using active materials, such as shape memory polymers (SMP)^19–22^, hydrogels^23^ or composites^24^, have provided the capability to print shape-evolving products, and thus adds time as the fourth dimension to the printed structures, or 4D printing. Among active materials, SMPs exhibit excellent recoverability, easy tailoring of properties. More recently, 3D printing SMPs become available, making them a good fit for fabricating active structural systems with complicated geometries.

**Figure 1:**
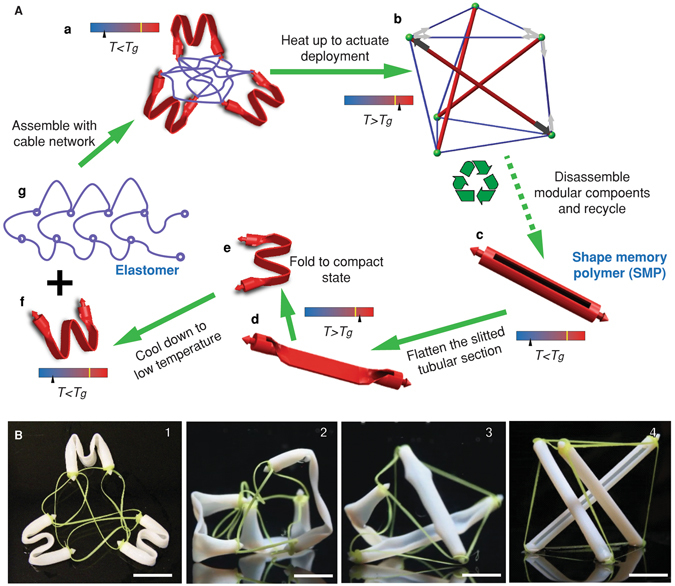
Procedure for creating an active tensegrity. Deployment of an active tensegrity is based on the shape recovery property of shape memory polymers (SMP). (**A**) Schematic of the overall concept and design. (a) The struts, which are programmed to compact shapes, are connected by a network of elastomer cables. (b) Upon heating, the recovery of the struts to their straight shapes leads to actuation of the structure to a 3D resilient tensegrity structure. To achieve this concept, (c) the struts are designed to have tubular shapes with longitudinal slit portions and are 3D printed using SMPs; (d,e) the SMP struts are folded into compact shapes at a temperature that is above the glass transition temperature (*T*_*g*_) of the SMP; (f) decreasing the temperature below *T*_*g*_ fixes the struts in the compact shapes, which are then assembled with the elastomer cables (g) according to the topology of the design to form a loose assembly (a); heating the assembly to a temperature above *T*_*g*_ leads the struts to their original shapes, and thus the constraints from the cables induce self-stress. As a consequence, a stable tensegrity structure is obtained. (**B**) The experimental result shows the deployment process. The scale bars represent 15 mm.

Here, we use 3D printed thermally responsive SMPs to create actively deployable tensegrities. Thanks to the aforementioned unique properties of tensegrity, our paradigm for creating self-deployable structures distinguishes itself from related attempts for reconfigurable structures^20–25^ in many aspects, such as superior volume expansion, design simplicity, resilience after deployment, and modularity. Figure 1A shows schematically the overall concept and the details of the design. The struts, which are made of SMP and are straight in their permanent shape, can be programmed into compact shapes. They are then connected by elastic cables (Fig. 1A–a). Once the assembly is heated, the struts recover their original straight shapes. However, because of constraints imposed by the cables, self-stresses are generated in both cables and struts, and the loosely connected struts and cables can stand up and form a fully functional 3D tensegrity structure (Fig. 1A–b).

## Results

### Design and Demonstration

To realize the aforementioned concept, we design the struts and cables and use 3D printing to implement our designs – Fig. 1A shows design details. The struts have tubular shapes with slit central portions so that they can be easily packed by bending (Fig. 1A–c). The two ends of the struts are designed with arrowheads to help mounting the cable network. Struts are printed by an acrylate-based photopolymer, named Verowhite, which is one of the model materials in our multimaterial 3D printer (Objet 260 Connex) and is a SMP with the glass transition temperature (*T*_*g*_) around 60 °C^19, 25^. The printed struts are then heated to a temperature (65 °C) above its *T*_*g*_ for programming. We first flatten the central portion (Fig. 1A–d) then bend it into a W-shape to enable favorable compaction (Fig. 1A–e). Finally, we lower the temperature to 10 °C and the struts are fixed in the W-shape (Fig. 1A–f). For the cables, because they form a continuous network^12, 14^, we design them (Fig. 1A–g) according to the structural topology and print them using an FFF (Fused Filament Fabrication) printer with Filaflex, which is a stretchable elastomeric filament material. The nodes in the cable network are designed with small holes so that they match the arrowheads of the struts. Finally, we attach the cable network with programmed struts (Fig. 1A–a). Up to this step, the tensegrity structure gains its topology but not its geometry; it is unconstrained in configuration and thus could be tightly packed into an arbitrary shape. We then increase the temperature to deploy the structure. Fig. 1B and Movie S1 show the deployment when the assemblage is thrown into a tank of hot water at ~65 °C. As the struts recover their original straight shapes, cables are stretched and self-stresses grow within the system. This renders “life” to our tensegrity, i.e. it stands up, to reach its designated geometry, resulting in a giant configurational change, although it had never been built to this shape before.

### Theoretical Analysis

**Figure 2:**
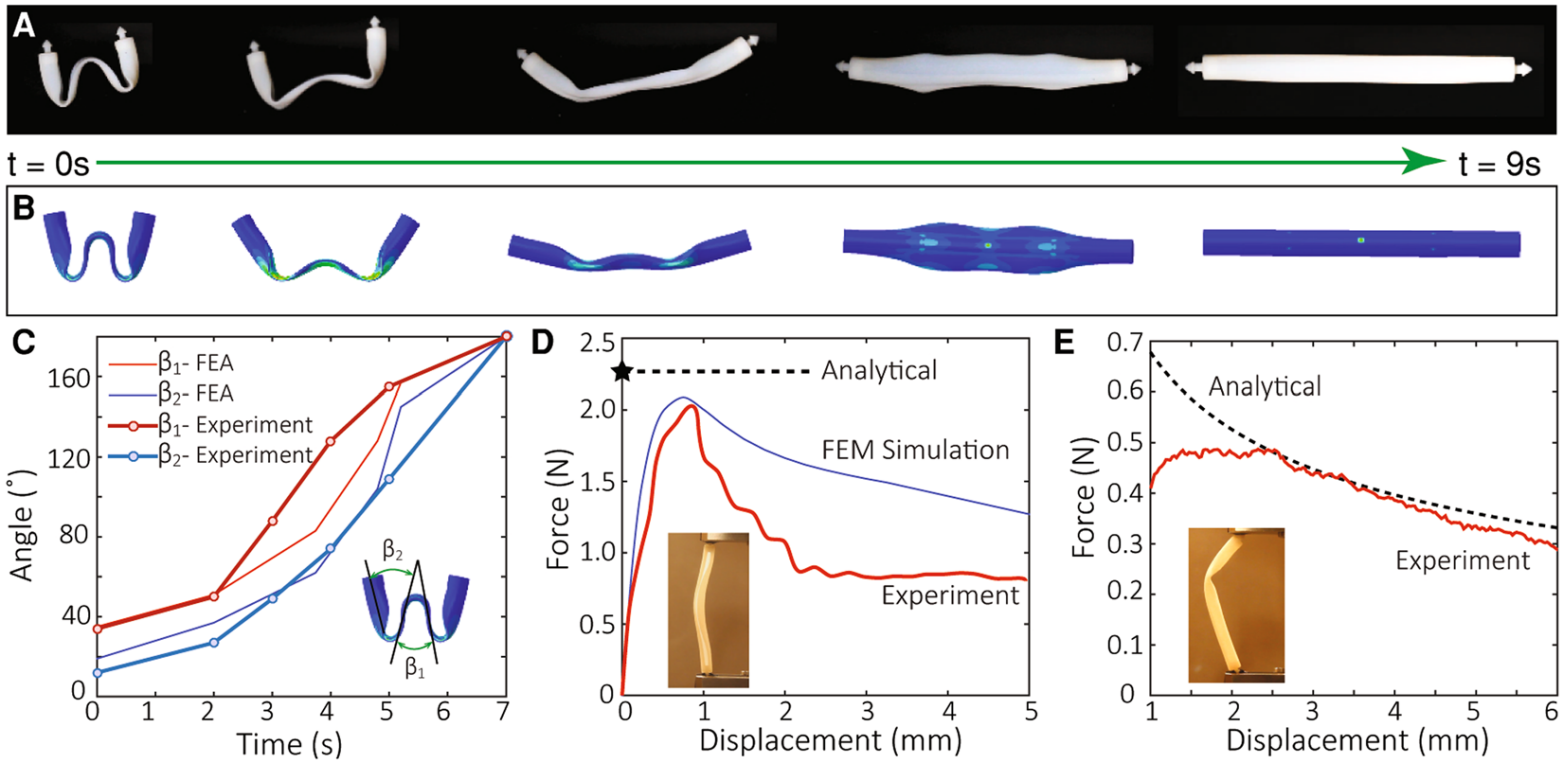
Properties of the slitted tubular struts via 3D printing. (**A**) The snapshot images of the free recovery sequence of a strut at 65 °C. (**B**) The predictions from the corresponding finite element method (FEM) simulation. (**C**) The opening angles of the strut during the free recovery and the comparison with the FEM simulation. The inset shows the definitions of the two opening angles. (**D**) The critical load of a single strut during uniaxial compression from the experiment and the FEM simulation. The inset shows the experimental setup. Considering the boundary condition in the experimental setup, a 0.75 effective length ratio gives the upper bound on the critical buckling load of a single strut. In practical designs, because the joints in our tensegrity are almost free in rotation, 1.0 effective length shall be used. (**E**) The critical load of a single strut during its recovery. Derivation for the analytical prediction is elaborated in *SI*. In the experiment, a small initial displacement (1 mm) is imposed to prevent the opened cross section from closing.

The mismatch between the initial lengths of the struts and cables is critical for determining the self-stresses, which in turn dictate if the deployment can be successful and the stiffness of the tensegrity is enough (see SI for details). In general, neither too small nor too large self-stresses can deploy the structure. This is because too small self-stresses would not provide enough stiffness to support the total weight, but too large self-stresses would prevent the strut from a full recovery. Therefore, it is important to design proper initial lengths. Toward this end, we conduct theoretical analysis of the self-stress generated during and after the strut recovery to gain insight (see SI). We also conduct finite element (FE) simulations to confirm our theoretical analysis. Fig. 2A and B show the comparison between the experiment and the FE simulation of the shape change of a strut during a free recovery (Movie S2). Fig. 2C shows the opening angles (defined in the inset) measured during the recovery. Overall, the FE simulation results match the experiments reasonably well. The difference mainly comes from the uncertainty from experimental measurement, which is a challenge due to the dynamic nature of the free recovery. To estimate the maximum self-stress beyond which a deployed strut will buckle, we conduct a compression test to measure the critical force (Fig. 2D). In addition, by using the effective length ratio of 0.75, the estimated buckling load derived using the Euler buckling criteria is close to those in the experiment and the FE simulation (see SI). The FE simulation shows relatively large deviation after the peak force is reached because instability occurs in the post-buckling regime. Nonetheless, the peak force is the most important design parameter. Fig. 2E compares the theoretical estimation and the experimental result of the critical force in the strut during the recovery, i.e. when the cross-section is open. The critical force for a strut during its recovery is typically smaller than the Euler buckling load after its recovery. On one hand, the different cross-sections lead to different elastic buckling loads﻿. On the other hand, the energy level of deformation state before the buckling is high, so the system quickly buckles into the post-buckling state, which is a more energetically favorable state. Nevertheless, during the recovery, the system is driven by its internal energy following a low energy path, which gives a lower force. This difference in the buckling force and the recovery force is beneficial; this is because the low recovery force makes the recovery relatively easy and the high buckling force can prevent the deployed tensegrity structure from buckling.

Hence, for our design of struts, the critical force shown in Fig. 2E determines whether a strut can successfully deploy when assembled in the tensegrity system. Based on the theoretical and FE analyses, we choose the initial lengths to be 70 mm for the strut, 49 mm for the horizontal cables, and 45 mm for the tilted cables (see SI). This design yields a maximum compression force in the struts to be 0.15 N, about half of the minimum critical force (i.e. the recovery force) of the strut. A compression test is applied on the final structure. By matching the initial stiffness with theoretical predictions, we can inversely determine the magnitude of the induced self-stresses. We achieve approximately a maximal compression in the struts around 0.20 N, larger than the designed value, but still less than the critical forces.

### Reduced Degree-of-Freedom Design and 3D Structures

**Figure 3:**
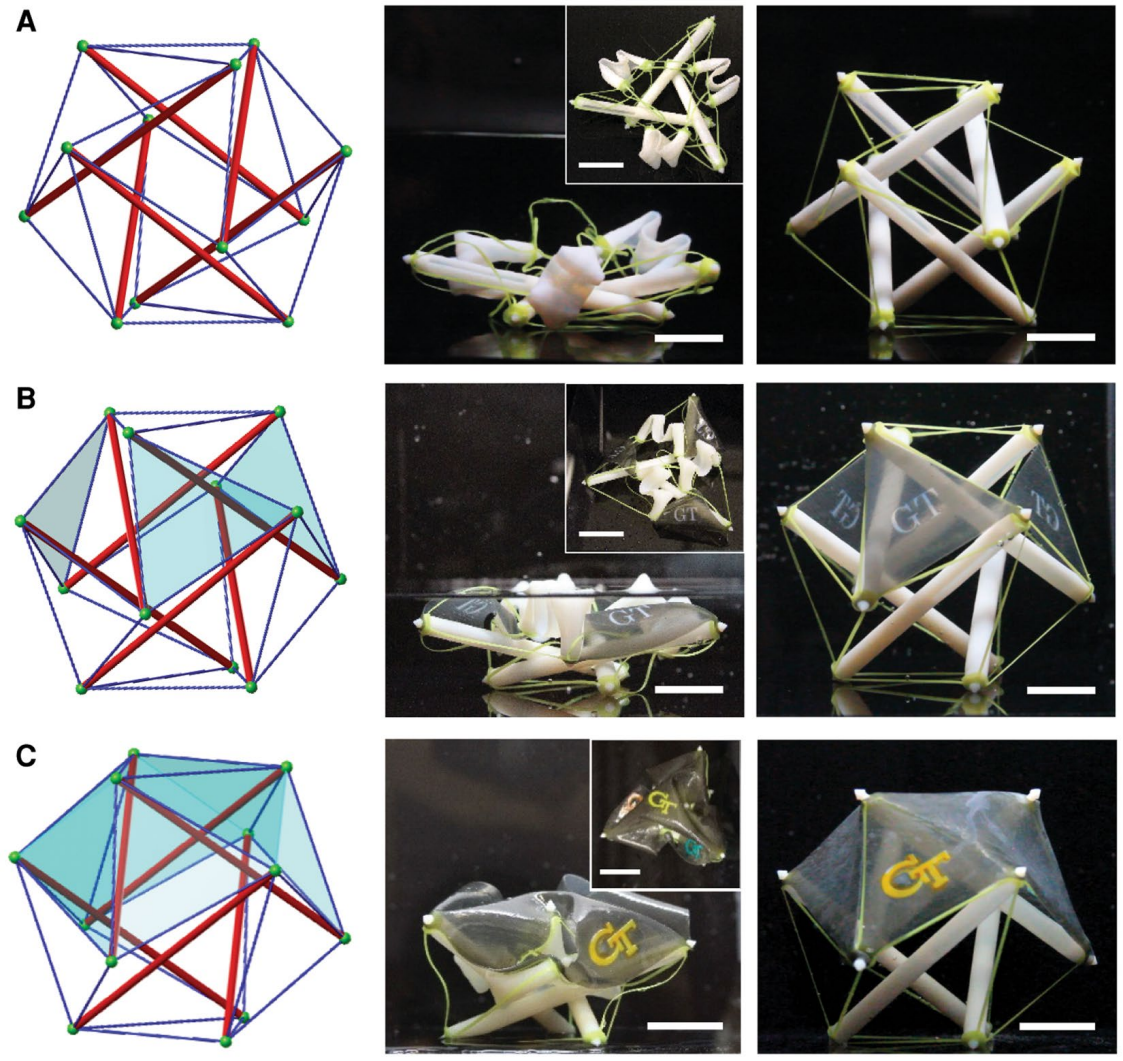
Deployment of 6-strut spherical tensegrity. (**A**) Deployment of a spherical tensegrity using the partial folding strategy to improve reliability of deployment. (**B**) Positioning of three discrete pieces of surfaces into space. (**C**) Deployment of a continuous surface supported by the active tensegrity to form a tent. The resultant structural system mimics the fundamental structure of vertebrates, with the membrane as skin, elastic cables as muscles, and relatively rigid struts as the skeleton (biomimetics). The scale bars represent 15 mm.

In our design, the cables are loose before deployment and the folded struts are free to move in space. Such excessive degrees-of-freedom may lead to incorrect positioning of struts and may create the risk of cable entanglement, or trap the structure at an undesirable configuration by (Fig. S7). To overcome such drawback, we reduce the degrees-of-freedom of the undeployed structure. One approach is to take advantage of the decoupled hierarchies and reduce the number of packed struts, i.e., leaving some struts straight. In this way, the tensegrity deployment becomes more deterministic, while the structure can still be stored in a compact state that occupies much less space than its deployed configuration. This design concept is illustrated by the 6-strut spherical tensegrity shown in Fig. 3A, where three of the struts are deprogrammed and are made partially solid to have an eccentric center of gravity, which stabilize the structure against gravity as it stands up. Such a design leads to successful deployment (Movie S3).

Our active tensegrity can be used to form 3D structures with surfaces that can serve as a platform to host functional devices. As a demonstration, we attached elastomer membranes (Fig. 3B and C) on the previous 6-strut tensegrity. On both discrete and continuous surfaces (Fig. 3B and C, respectively), we printed the “GT” (Georgia Tech) logo; it is not hard to imagine that one can print electronic circuits, to take advantage of the gigantic shape-change and to enable functionalities of the structure. Movie S4 and S5 show the deployment processes. The configuration of the deployed surfaces depends on the base tensegrity. With some state-of-art form-finding approaches for tensegrity^26–28^, we can generate space covering surfaces of almost any geometry. In addition, the attached surfaces increase the reliability of the deployment, as they provide additional constraints and reduce arbitrariness during the deployment.

### Sequential Deployment

**Figure 4:**
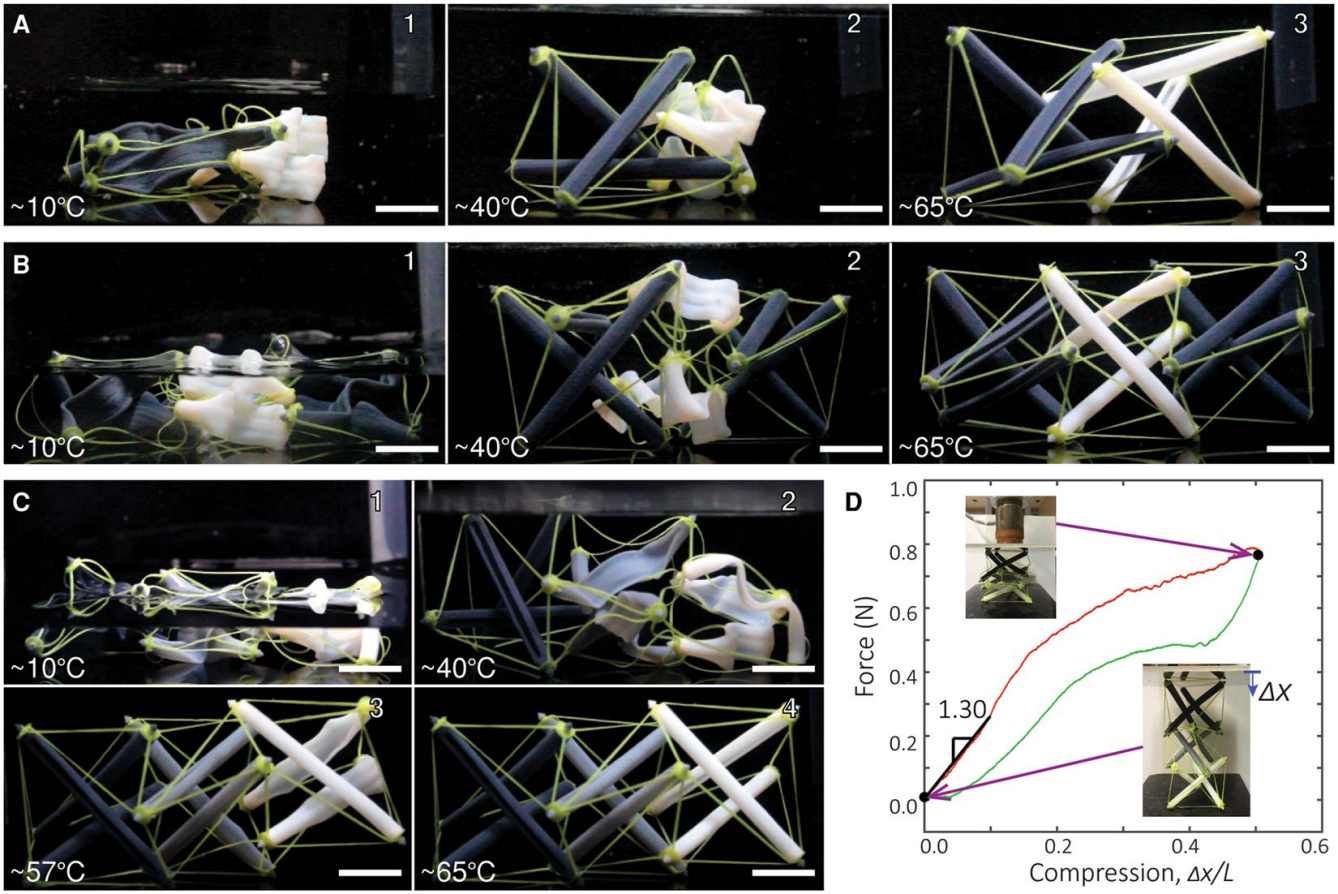
Programmed deployment of layered tensegrity structures. (**A**) Programmed deployment sequence of the 2-layer tensegrity using 2 different SMPs for the struts [DM-1 (the dark grey material) and BM (the white material)]. (**B**) Programmed deployment sequence of the 3-layer tensegrity using 2 different SMPs for the struts [DM-1 (the dark grey material) and BM (the white material)]. The two end layers have struts made with SMP of a lower *T*_*g*_ than the middle layer. (**C**) Programmed deployment sequence of the 3-layer tensegrity using 3 different SMPs for the struts. The three SMPs have increasing *T*_*g*_’s from left to right. The darker the color, the lower the *T*_*g*_. (**D**) Compression test of the deployed 3-layer tensegrity made with 3 different SMPs. The test reveals a maximum compression in the struts around 0.12 N. The scale bars represent 15 mm.

The development of digital materials in 3D printing allows us to print parts using polymers with different *T*_*g*_’s, thus offering different shape memory characteristics that permit sequential shape changes^20, 29^. We take advantage of the digital SMPs and program the deployment sequence to further pursue complex tensegrities in a controlled manner. Here, we choose three SMPs: DM-1 with *T*_*g*_ around 37 °C; DM-2 with *T*_*g*_ around 57 °C; and the SMP used in the above (Verowhite, termed as BM here) with *T*_*g*_ around 60 °C (see SI). We first create one 2-layer prismatic tower tensegrity (Fig. 4A), and one 3-layer tensegrity^1, 30^ (Fig. 4B), by using DM-1 and BM, to demonstrate the capability of the programmed deployment. The struts with different materials are programmed in the same manner as shown in Fig. 1. They are then assembled with the elastomer cable networks. Fig. 4A–1 and B–1 show the unactuated shapes of the structures. As there are no self-stresses, they lay on the ground. To activate the structure, we first increase the temperature to 40 °C by submerging the structure in a hot water bath. As shown in Fig. 4A–2 and B–2, the struts made by DM-1 recovered first, forming partially deployed tensegrity structures, with the right and middle parts not activated in Fig. 4A–2 and B–2, respectively. Finally, we increase the temperature to 65 °C to deploy the struts made with the BM, as shown in Fig. 4A–3 and B–3. The deployments of the two tensegrities are recorded in Movies S6 and S7.

To further demonstrate control over the deployment sequence, we prepare a three-layer structure with DM-1 DM-2, and BM (Fig. 4C). To deploy the structure, we increase the temperature in three steps: first to 40 °C, then to 57 °C and finally to 65 °C. Fig. 4C–2 to C–4 shows the sequential deployment (Movie S8). Because the glass transition temperatures of DM-2 and BM are close, the distinction between the actuations of the middle layer and the right layer are not very clear; better distinction can be achieved if more digital materials were available with more distinguishable *T*_*g*_s.

### Mechanical Behaviors of Tensegrity

The obtained tensegrity structures allow elastic deformation to a significant amount of magnitude without fracture or yielding. Figure 4D shows a compression test of the 3-layer structure in Fig. 4C. Since the stiffness of the cables is much lower than the struts (see SI), the global deformation of the tensegrity is mainly carried by local deformation of the elastomer cables. The plateau in the loading curve and the small dip in the unloading curve in Fig. 4D are caused by the inherent multi-stability feature of this tensegrity design. By matching the initial stiffness with theoretical predictions (Fig. S1), we can inversely approximate the magnitude of induced self-stresses. The calculation and estimation for other tensegrity structures in Fig. 4 can be found in Fig. S8.

## Discussion

The tensegrity in our design paradigm consists of *two hierarchies*: the *first hierarchy* is the compaction and recovery of individual struts; the *second hierarchy* is the final geometry of the tensegrity, i.e. the global structure. Therefore, the final configurational change is composed of both material-induced shape change and topology-induced shape change. The *second hierarchy* amplifies the *first hierarchy* to achieve gigantic volume expansions. Furthermore, these two hierarchies are decoupled, i.e. the final tensegrity does not depend on how we design and compact the strut (the first hierarchy). Therefore, other designs of the struts, such as different cross-section shapes or programed shapes can be used. In this paper, our design of each strut is inspired by the storable tubular extendable member (STEM)^31^ usually used on satellites. Such a design provides a relatively high critical force after recovery. The slit design enables favorable deployments. However, this is not the only design alternative and thus one can design the strut based on other considerations^20, 23, 24^. As shown above, we avoid specialized design and dedicated fabrication for every new active structure, but can apply components of the same design to create different structures by varying combinations, in a way similar to the LEGO toy, which opens a new venue that allows for quick fabrication of 3D active structures through modular designs. We can also recycle the struts to save material and reduce waste.

In retrospect, we create a method for realizing active tensegrity by combining 3D printing with actuation to deploy 3D structures that respond to environmental stimuli. Our paradigm of active tensegrity is unique and novel as it integrates the complementary features of tensegrity structures and smart materials, merging the frontiers of structural mechanics and material science. The intriguing properties of tensegrity allows the active deployment to have two decoupled hierarchies: programming the SMP struts into compact shapes, and the topology of the actual tensegrity. Such a decoupling strategy leads to gigantic shape change, allows for modular design, and provides rich programmability and tunability. The struts are allowed to have others shapes and be programmed into a compact shapes so that they can be assembled with the elastomer cables according to the topology of the tensegrity. The active tensegrity structures can be programmed to deploy in a sequential fashion by differentiating the glass transition temperatures of the SMPs used for the struts. Further enrichment includes, for example, using shape memory composites^22^ to achieve finer control of shape change, or using materials such as hydrogels^23, 24^ to design the structure to respond to different types of environmental stimuli. In addition, surfaces, which could be used as a platform for integrating functionality, can be attached to the nodes in the tensegrity to enable active devices with dramatic property changes. Therefore, our paradigm of active tensegrity offers a platform for generic devices/applications that can benefit from the gigantic shape changes reported in the present research. With unique properties of tensegrity and remote controllable actuation by temperature, we can foresee the great potential of active tensegrity in various applications. For example, tensegrity structures have been successfully exploited as deployable antenna and reflectors on satellites, for example, contractible reflector for a small satellite that can be packaged within an envelope^32, 33^. Another application is the tensegrity robot for locomotion and duct systems^34, 35^. In addition, Carpentieri *et al*. recently provides a method to use the minimal mass deployable tensegrity for solar energy harvesting on water canals^36^. These traditional applications of tensegrity usually need mechanical drivers to deploy. Now, empowered by SMP, the active tensegrity structure is self-deployable, with the capability to adapt automatically to environmental changes. The active tensegrity may also be applied for biomedical purpose, such as stent^7, 37, 38^. A stent is a type of flexible tubular device for minimally invasive surgery. It is capable of being folded into small dimensions and then deployed to open up a blocked lumen. The active tensegrity could be suitable for self-deployable stent which deploys under human body temperature once inserted. There are various tensegrity designs that approximate tubular shapes^12^. In addition, as we showed in this paper (Fig. 4D, Fig. S8), deployed active tensegrity structures have great resilience to undergo large elastic deformations, which is a desired feature for biomedical devices so that the stent can also adapt to the deformation of human tissues.

## Methods

### Sample fabrication

The slitted tubular struts were fabricated using an Objet 3D printer (Objet 260 Connex, StrataSys Inc, Eden Prairie, MN, USA) in digital material mode using the PolyJet technology. The printer can combine two base materials, using pre-determined ratios to make the so-called digital materials. The digital materials differ in mechanical and thermal properties. The curable liquid photopolymer was jetted onto the build tray and then cured by UV polymerization. The three digital materials used in this paper are Verowhite plus, DM9895 (DM-1) and DM8530 (DM-2) in Stratasys material library. The cables were fabricated using the Fused Filament Fabrication (FFF) technology on a HYREL 3D Printer (System 30 M, Hyrel 3D Inc, Norcross, GA, USA). A rubbery material named Filaflex (Recreus, Elda, Spain) was used, which is a thermoplastic elastomer base polyurethane. The extruder was especially equipped with a dual drive system to fulfill the task of printing flexible filaments. The filament was melted at ~232 °C and deposited through a nozzle of 500 µm diameter onto the tray. The cable nets were printed by two passes of reversed orientation. The extrusion paths were optimized to ensure the quality of the printing.

### Deployment control

A water temperature control system was built, which includes a glass water tank, a DC hot water pump, a water heater, an electrical thermometer, and plastic tubes. The tank held some cold water (~10 °C) at the beginning of each experiment. The level of the cold water submerged the undeployed tensegrity assemblies. To activate the deployment, hot water (~95 °C) was pumped from the water heater into the tank to increase the temperature of the cold water, which is monitored by an electrical thermometer. In the programmed deployment test, we stopped injecting hot water once the water reached the desired temperature. After the whole tensegrity deployed, we were able to drain the water from the tank.

### Compression tests of the deployed tensegrity

We performed the compression tests of the deployed tensegrity structures using an electromechanical universal material test machine (MTS Criterion® Series 40, Eden Prairie, MN, USA) at room temperature (~25 °C). The deployed tensegrity was placed on a flat stage and then compressed by another flat plate mounted to the load cell. The stage and plate were lubricated to reduce friction. The compression loading rate was set to be 0.2 mm/s. The forces and displacements were recorded at a 10 Hz sampling rate, and a load cycle was performed. The unloading commenced when the global deformation (compression) reached half the height of the tensegrity.

## Electronic supplementary material

Supplementary Information

Video 1

Video 2

Video 3

Video 4

Video 5

Video 6

Video 7

Video 8
