# Supplementary material for: Programmable Deployment of Tensegrity Structures by Stimulus-Responsive Polymers
Source: Sci Rep. 2017 Jun 14;7:3511. doi: 10.1038/s41598-017-03412-6 (PMC5471187; doi:10.1038/s41598-017-03412-6)
Supplement: Supplementary file 1 — Supplementary Information [file 41598_2017_3412_MOESM1_ESM.pdf]

Supporting Information

Programmable Deployment of Tensegrity Structures by Stimulus-Responsive Polymers

Ke Liu[1], Jiangtao Wu[1], Glaucio H. Paulino, H. Jerry Qi**

[1]These authors (K. Liu and J. Wu) contributed equally to this work.

K. Liu, Prof. G. H. Paulino
School of Civil and Environmental Engineering, Georgia Institute of Technology
5142B Jesse W. Mason Building, 790 Atlantic Drive NW, Atlanta, GA 30332, USA
E-mail: paulino@gatech.edu
J. Wu, Prof. H. J. Qi
George W. Woodruff School of Mechanical Engineering, Georgia Institute of Technology
801 Ferst Drive MRDC 4104, Atlanta, GA 30332, USA
E-mail: qih@me.gatech.edu

Materials and Methods

Material characterization: A dynamic mechanical analysis (DMA) machine (Model Q800, TA Instruments Inc, New Castle, DE, USA) was used to characterize the mechanical and thermomechanical properties of the materials. The viscoelastic properties of the printed SMPs were measured in the film tension mode. The material samples (dimension 10mm×3mm×1mm) were first heated to 90°C on the DMA machine and stabilized for 10 minutes to reach thermal equilibrium. A preload of 0.001N was applied to straighten the samples. During the DMA tests, the strain of the samples was controlled to oscillate at a frequency of 1 Hz with a peak strain amplitude of 0.1%. Meanwhile, the temperature decreased from 90°C to 0°C with a rate of 2°C/min. The glass transition temperature Tg is identified by the temperature when the viscoelastic loss tangent (tanδ) reaches its peak value. The Tg's of the three strut materials are 60°C (BM: Verowhite plus), 37°C (DM-1: DM9895), and 57°C (DM-2: DM8530).

The stress-strain behavior of the cable material was tested in controlled force mode on the DMA machine at room temperature (~25°C). A complete load cycle was performed at a very low speed (quasi-static) on a sample with dimensions 10mm×0.9mm×0.25mm. The printed sample was stretched to 3.1 MPa at loading rate of 0.5 MPa/min and then unloaded. The initial tangent elastic modulus was determined to be 16.43MPa from the stress strain curves (see Figure S5).

Supporting Text

S1. Analysis of self-stressed tensegrity structures

Based on the design, linear analysis of self-stressed tensegrity helps us to correlate the initial tangent stiffness of the tensegrity structure to its self-stress level. Considering the small strain due to self-stress, we treat the strut material as linear elastic and take the initial modulus for the calculation to simplify the design process. The governing equation takes the form of a linear equation $\mathbf{Ku} = \mathbf{F}$, where $\mathbf{K}$ is the stiffness matrix, $\mathbf{u}$ contains the nodal displacements, and $\mathbf{F}$ contains the applied forces. Due to the self-stress, the stiffness matrix for a tensegrity structure is different from a normal truss structure[1,2]. The major difference is the additional contribution of the geometrical stiffness matrix $\mathbf{K}_G$. Thus the tangent stiffness matrix takes the form:

$$\mathbf{K} = \mathbf{K}_E + \mathbf{K}_G, \tag{S1}$$

where, $\mathbf{K}_E$ is the linear stiffness matrix. For completeness, we summarize the derivation of $\mathbf{K}_G$ here. Assume that for a member (either a cable or strut) i, its two nodes, length and self-stress induced force are a, b, L_i and T_i, respectively. We define the components of a connectivity matrix $\mathbf{C}$ as:

$$C_{ij} = \begin{cases} 1, & \text{if member } i \text{ is connected to node } j \text{, and } j = a \\ -1, & \text{if member } i \text{ is connected to node } j \text{, and } j = b \\ 0, & \text{otherwise} \end{cases}. \tag{S2}$$

We also define a diagonal matrix $\mathbf{Q}$ such that:

$$Q_{ii} = T_i / L_i \,. \tag{S3}$$

The ratio T_i/L_i is known as the force density[2]. Let $\overline{\mathbf{T}}$ be the normalized self-stress induced force vector with maximum compression in struts equal to 1. Denoting γ as a scaling factor (which equals to the maximum compressive force in struts), we can rewrite $\mathbf{Q}$ as:

$$\mathbf{Q} = \gamma \overline{\mathbf{Q}}, \tag{S4}$$

where $\overline{Q_{ii}} = \overline{T_i} / L_i$. Because $\overline{\mathbf{T}}$ is an intrinsic property of a tensegrity design, it is a constant vector. Thus, we can write the so-called force density matrix [2] as:

$$\mathbf{E} = \mathbf{C}^T \mathbf{Q} \mathbf{C} = \gamma \mathbf{C}^T \overline{\mathbf{Q}} \mathbf{C} \,. \tag{S5}$$

Since the strains of the members caused by the self-stress are small, the geometric stiffness matrix can be expressed as:

$$\mathbf{K}_G = \mathbf{E} \otimes \mathbf{I}_{3\times 3} = \gamma \overline{\mathbf{K}_G} \,. \tag{S6}$$

Finally, with the contribution of the linear stiffness matrix, the stiffness matrix of the tensegrity structure can be approximated for small deformation as:

$$\mathbf{K}(\gamma) = \mathbf{K}_E + \gamma \overline{\mathbf{K}_G} \,. \tag{S7}$$

From the above derivation, we can see that the stiffness matrix of a tensegrity is a function of its self-stress level γ. We find that the higher the self-stress, the stiffer the tensegrity. Using this equation, we can find the relationship between the initial tangent modulus of a tensegrity under global uniaxial compression and the self-stress level γ. The initial tangent modulus is the ratio of the applied force over the compression magnitude (in terms of displacement). Figure S1 plots the curve of initial tangent modulus versus self-stress level, based on the material properties for 3 tensegrity designs. The tangent modulus shown here is calculated using the non-dimensional

displacements, which is the downward compression displacements normalized by the heights of the tensegrity designs, and thus, the unit of the tangent modulus is in Newton (N). The two 3-layer tensegrities (with different materials) yield almost identical curves, so only one is plotted for clarity. This curve does not start from (0,0) because the 3-layer tensegrity is kinematically determinate, thus its stiffness matrix is not singular when there is no prestress ($\mathbf{K}(0) = \mathbf{K}_E$). According to the experimental compression tests of the active tensegrities, we can approximate the initial tangent modulus of a tensegrity. Then, based on the curves shown in Figure S1, we can inversely estimate how much self-stress we have successfully applied to the active tensegrity.

S2. The two critical loads for the slitted tubular struts

From the previous section, we can see that the (initial) stiffness of a tensegrity structure depends on the self-stress level. However, the achievable self-stress level of an active tensegrity is not arbitrary, as it is determined by two critical factors. *The first factor is that the compression on struts should not prevent their full recovery.* In the final stage of deployment, some SMP struts in the active tensegrity will be subject to compression before full recovery, with their tubular cross-section still open. *The second critical factor is that, after deployment, the struts should not buckle under the self-stress compression.* If the struts buckle, then the tensegrity will lose some self-stress and cannot completely reach the designated shape. In the following, we will derive analytical estimations of these two critical strut loads. We first compute the critical force during the recovery, when the tubular cross section of a strut is open, as shown in Figure S2a. We make the following assumptions: *(1) a tube can be analyzed using shell theory because the thickness is relatively small; (2) the mid-surface is subject to isometric deformation; (3) the static behavior of the SMP can be regarded as elastic when the temperature is fixed and the strain is relatively small.* The meaning of the symbols used in the derivation is illustrated in

Figure S2. Therefore, supposing that the changes in the curvatures along the two principle directions are ($-1/r,\ 1/R$), we can write the total strain energy at the bending region as[3]:

$$U_B = \frac{D\alpha R\psi}{2}\left(\frac{1}{r}+\frac{r}{R^2}-\frac{2\nu}{R}\right). \tag{S8}$$

The symbol D denotes the flexural rigidity, defined as:

$$D = \frac{Et^3}{12\left(1-\nu\right)}, \tag{S9}$$

where E is Young's modulus and v is Poisson's ratio. The value of r is determined when U_B is minimized[3]. Therefore, $r=R$. Then the bending moment is calculated as:

$$M = \frac{\partial U_B}{\partial \psi} = D\alpha\left(1-\nu\right). \tag{S10}$$

At the final stage of the strut's recovery, a single kink about a quarter from the end of a strut is usually observed (see Movie S3). Thus, we can draw the shape schematically as shown in Figure S2c. The regions that are not opened are much stiffer than the bending region. Therefore, we may treat those regions as rigid. Notice that,

$$\lambda L\sin\theta_1 = (1-\lambda)L\sin\theta_2. \tag{S11}$$

If the two applied forces are aligned along the same line, then equilibrium is obtained as:

$$M = F_{cr}\left(\lambda L\sin\theta_1 + R\cos\theta_1\right). \tag{S11}$$

Thus the critical force can be calculated by:

$$F_{cr} = \frac{M}{\lambda L\sin\theta_1 + R\cos\theta_1} \geq \frac{Et^3\alpha}{12(\lambda L\theta_1 + R)},\ (0<\theta_1<\pi/2). \tag{S11}$$

In our case, the typical value for λ is around 0.25. The angle θ_1 can be computed from the deformed length of the strut ($\lambda L\cos\theta_1 + (1-\lambda)L\cos\theta_2$). The equality holds when θ_1 is small. The later expression is used because it is simple and conservative. The derivation requires a portion with fully opened cross section along the strut (which forms a "kink"), thus it is not accurate

when the strut is almost straight (i.e. θ_1 becomes very small), because in reality the opened cross section starts to enclose before the strut recovers to straight, so the deformation mode no longer has a "kink".

The critical load before the buckling of the struts after deployment is given by the Euler buckling formula[4],

$$F_{buckling} = \frac{\pi^2 EI_{min}}{L_{eff}^2}. \tag{S12}$$

The effective length L_{eff} depends on the boundary conditions of the strut. In the compression tests, the fixture of the sample constrains the free rotation at the two ends, resulting in an effective length around $0.75L$. However, in the tensegrity, the two ends are assumed to be pinned, and thus $L_{eff} = L$. The minimum static moment of inertia I_{min} is determined to be the static moment of inertia of the *X-X* axis at the geometric centroid *GC*, which is denoted as $I_{GC\text{-}XX}$,

$$I_{GC-XX} = R^3 t\left[\frac{\alpha}{2} + (\alpha - \pi)\frac{\sin^2(\alpha/2)}{(\alpha/2)^2} + \frac{\sin\alpha}{2}\right]. \tag{S13}$$

We note that, in the experiment, the struts are not loaded at the geometric center (*GC*) of the cross section. Instead, the compressive forces are loaded at point O (at the center of the mid-surface circle). As a consequence, the actual critical buckling force will be lower than the estimation, since the buckling mode involves a combination of bending and twisting.

S3. Design of cables

As explained in our paper, the self-stress in the tensegrity is induced by prescribed length differences between cables and struts. We assume that after successful deployment, the struts become straight and their deformation under compression is negligible (recall that the struts are much stiffer than the cables). Therefore, we control the level of self-stress magnitude by

manipulating the initial length of cables. We did this for two reasons. First, we do not want the initial length of cables to be too long so that the deployed tensegrity cannot gain enough self-stress to become stable and stiff. Second, the initial lengths of cables should not be so short that the struts cannot recover during deployment or stay straight after deployment, due to the excessive self-stress magnitude.

Suppose that the desired self-stress level is γ and member *i* is a cable. Given the normalized force vector $\bar{\mathbf{T}}$ (as defined in Section S1), we can determine the initial length of a cable as:

$$ {}_0 = \frac{{}_d - 2\delta}{(\gamma \bar{T}_i)/(AE_c)+1} + 2\delta . \quad \text{(S14)} $$

In this equation, ${}_0$ denotes the initial length, ${}_d$ is the design length of the cables which is pulled from the geometry of the tensegrity design, $\gamma \bar{T}_i$ is the desired tension in the cable, A is the cross-sectional area of the cable, and δ is the ineffective length at each end of a cable which changes very little. Considering the contact angles of cables and struts, δ is generally 1.4~3 times the distance *d* shown in Figure S3. Typically, the force is small, and we can assume linear behavior for the cables. Hence the initial elastic modulus E_c is used.

Such calculation provides an approximate guide for determining the initial lengths of cables based on the value of γ, which needs to be greater than 0, but less than the minimum critical load of the strut. In reality, the control of the self-stress level and final geometry will not be precise due to many practical factors, for example: the twisting of cables, the plasticity of the cable material, the printing accuracy, and the entanglement of the cables near the joints. In some cases, adjustment based on the experimental results is needed, especially for tensegrity designs with complex geometries.

S4. Detailed experimental analysis

The shape recovery behavior of the strut comes from the viscoelastic properties of the SMPs. The DMA tests are performed to investigate the viscoelastic properties of the printed strut materials. The storage modulus and loss tangent tan δ vs. temperature plots of the printed three strut materials are shown in Figure S4.

The Filaflex material exhibits rubber-like viscoelastic properties at room temperature. Uniaxial tension tests are performed to investigate the mechanical properties of the printed Filaflex material. The stress vs. strain curve is shown in Figure S5. The specimen occupies the same cross section as the cables, which is a rectangle of 250μm-thick and 920μm-wide. The uniaxial stretch is up to ~40% of the initial length.

S5. Constitutive model for the SMP

The multi-branch model is used to describe the viscoelastic properties of the printed SMP materials. In this model, one elastic equilibrium branch and several thermo-viscoelastic non-equilibrium branches are arranged in parallel. The non-equilibrium branch is described by the Maxwell element, represented by a viscous damper and an elastic spring connected in series. The total stress of the material can be expressed as:

$$\sigma_{total} = \sigma_{Eq} + \sum_{m=1}^{n} \sigma_{non}^{m} = E_{Eq} e + \sum_{m=1}^{n} E_{non}^{m} \int_0^t \frac{\partial e}{\partial s} \exp\left[-\int_s^t \frac{dt'}{\tau_m(T)} \right] ds, \quad \text{(S15)}$$

where E_{Eq} is the Young's modulus of the equilibrium branch and both E_{non}^{m} and τ_m are the Young's modulus and temperature dependent relaxation time of the *m*-th non-equilibrium branch. To consider the temperature effects, the time temperature superposition principle (TTSP) is used. The relaxation time τ_m at temperature T can be calculated using the relaxation time τ_m^R at the reference temperature, given by:

$$\tau_m(T) = a^{Shift}(T)\tau_m^R, \tag{S16}$$

where $a^{shift}(T)$ is the time temperature superposition shifting factor. According to O'Connell and McKenna[5], the shifting factors can be calculated by combining the Williams-Landel-Ferry (WLF) equation [6] and the Arrhenius-type equation [7]. If the temperature is higher than the reference temperature, the shifting factor can be expressed using the WLF equation:

$$\log\left[a^{shift}(T)\right] = -\frac{C_1(T - T_{ref})}{C_2 + (T - T_{ref})}, T > T_{ref}. \tag{S17}$$

The parameters C_1, C_2 and T_{ref} are material parameters to be characterized by experiments. We denote A, F_c, and k^{Boltz} as the material constant, configurational energy, and Boltzmann's constant, respectively. When the temperature is lower than the reference temperature T_{ref}, the shifting factor is expressed by the Arrhenius-type equation:

$$\ln\left[a^{shift}(T)\right] = -\frac{AF_c}{k^{Boltz}}\left(\frac{1}{T} - \frac{1}{T_{ref}}\right), T < T_{ref}. \tag{S18}$$

The parameters including E_{eq}, E_m^{non}, τ_m^R, C_1, C_2 and AF_c/k^{Boltz} are determined from the DMA tests. The storage modulus at high temperature (90˚C for BM, 65˚C for DM-1, 85˚C for DM-2) is the equilibrium modulus E_{eq} for each of the materials. For the multi-branch model, the temperature dependent storage modulus $E_s(T)$, loss modulus $E_l(T)$ and loss tangent $\tan\delta(T)$ can be respectively computed by:

$$E_s(T) = E_{eq} + \sum_{m=1}^{n} \frac{E_m^{non}\omega^2\left[\tau_m(T)\right]^2}{1 + \omega^2\left[\tau_m(T)\right]^2}, \tag{S19a}$$

$$E_l(T) = \sum_{m=1}^{n} \frac{E_m^{non}\omega\tau_m(T)}{1 + \omega^2\left[\tau_m(T)\right]^2}, \tag{S19b}$$

$$\tan\delta\left(T\right)=\frac{E_l\left(T\right)}{E_s\left(T\right)}. \quad \text{(S19c)}$$

The symbol ω denotes the test frequency. By employing a nonlinear regression software [8,9], the parameters E_m^{non}, τ_m^R, C_1, C_2 and AF_c/k can be determined by fitting the $\tan\delta$ and storage modulus from experimental DMA tests. The material parameters used in this paper are provided in Table S1.

To show the capability of this model, the comparison of the DMA curves between the experiment and the simulation are shown in Figure S6. We can see that the multi-branch model explains the thermomechanical behavior of the printed strut materials in the temperature range used for programming and actuation processes.

S6. Finite element analysis

The recovery process and mechanical properties of struts are modeled using the FEA software ABAQUS (Simulia, Providence, RI, USA). The hybrid C3D8RHT element is used. We implement the multi-branch model based on Prony's series, which is defined as:

$$G(t)=G_{Eq}+\sum_{m=1}^{n}G_m e^{-t/\tau_m}, \quad \text{(S20)}$$

where G is the total shear modulus, G_{Eq} and G_m are the shear modulus of the equilibrium branch and m-th non-equilibrium branches. Applying the incompressible condition, the shear modulus G is calculated as $G_m=E_m/3$, where E_m is the elastic modulus from the multi-branch model. The material parameters for the multi branch model are elaborated upon in Section S5. To apply the temperature effects, the shift factors are calculated using the WLF equation and Arrhenius-type equation[6,7]. The UTRS subroutine is used to implement the WLF equation and Arrhenius-type equation.

Considering the symmetry of the strut and boundary conditions, only 1/4 of a strut is used for simulation of free recovery. The slit of the strut is first opened into a nearly flat configuration in the middle part of the strut at 65°C, which is above the T_g of the BM (Verowhite). The pressure used to open the slit is applied on the inner surface of the slit near the opening. After the slit is opened, we fix the middle section of the strut (one end in the 1/4 model) and add a pressure load on the end of the strut and in the transverse direction of the strut to bend it into a "U"-shape. To further deform the strut into the "W"-shape, we fix the 1/4 section of the strut and apply pressure at the end in the opposite direction of the previous step. After the deformation process is finished, we cool the temperature to 25°C, at which the material is in a glassy state. Then all the external loading and constraints are removed, and the deformed shape of the strut is "frozen" due to viscoelasticity. To simulate the recovery process, the temperature is increased to 65°C. The recovery process by the simulation is compared with the experimental results, as shown in Figure 2.

The strut under compression is also modeled to determine the after-recovery critical force ($F_{buckling}$). In this simulation, the whole strut is modeled to consider asymmetric deformation modes. We impose an ambient temperature of 65°C. One end of the strut is pinned in directions *x, y, z* within the central zone (radius of 1mm), creating a partially fixed end. At the other end, the center zone is pinned in *x, y* directions (partially fixed), and a displacement load of rate 0.25mm/s is applied in the -*z* direction. This boundary condition is similar to the case of the strut compression experiment, but more restrictive than the actual boundary condition as embedded in the tensegrity structures.

A similar procedure can be applied to predict the mechanical performance of struts made with various SMPs.

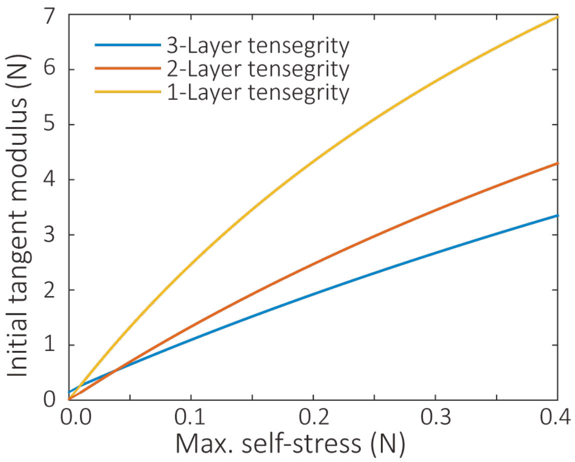

Figure S1. Initial tangent modulus vs. maximum self-stress forces in struts.

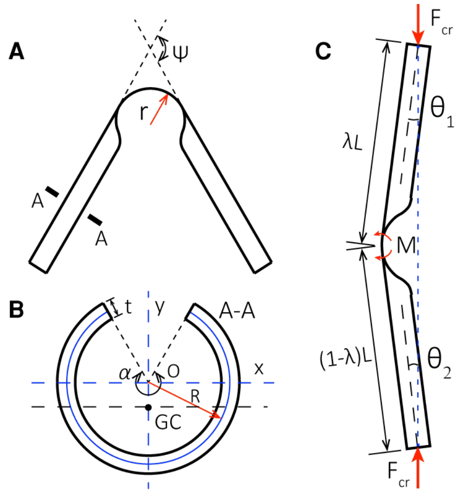

Figure S2 (A) Schematic of a folded strut with opened cross section. (B) Cross section (A-A) of the struts. (C) Sketch of the critical scenario in the recovery of struts (during the deployment of an active tensegrity), based on observations from the experiments.

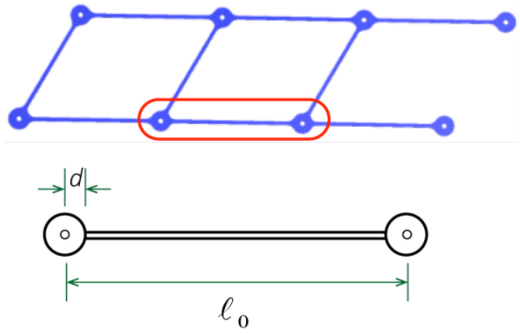

Figure S3 Schematic of the cable network design.

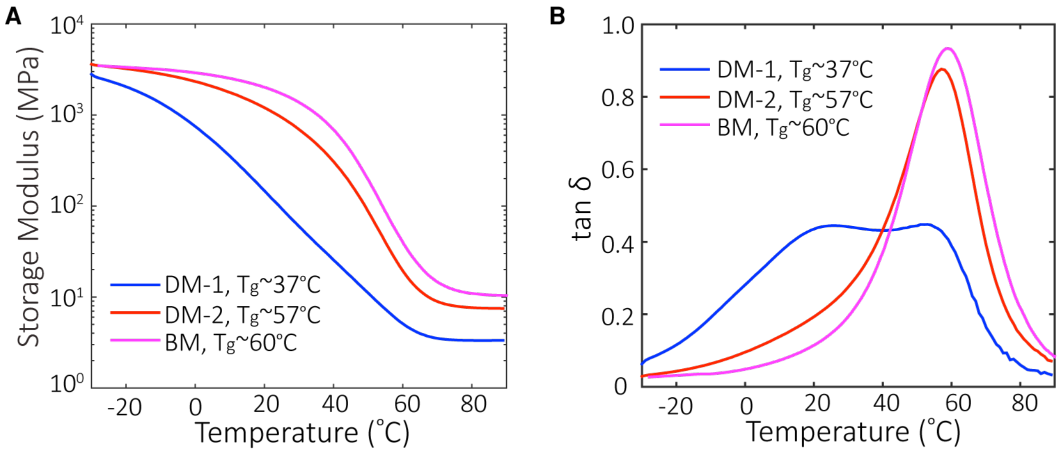

Figure S4 (A) Storage modulus vs. temperature curves for three SMPs. (B) Loss tangent tan δ vs. temperature curve.

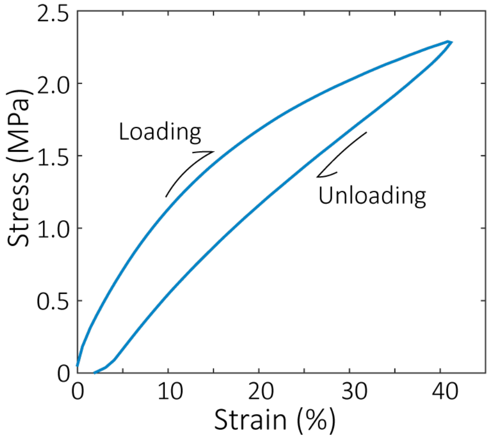

Figure S5 The stress-strain curve of Filaflex material at room temperature (~25°C).

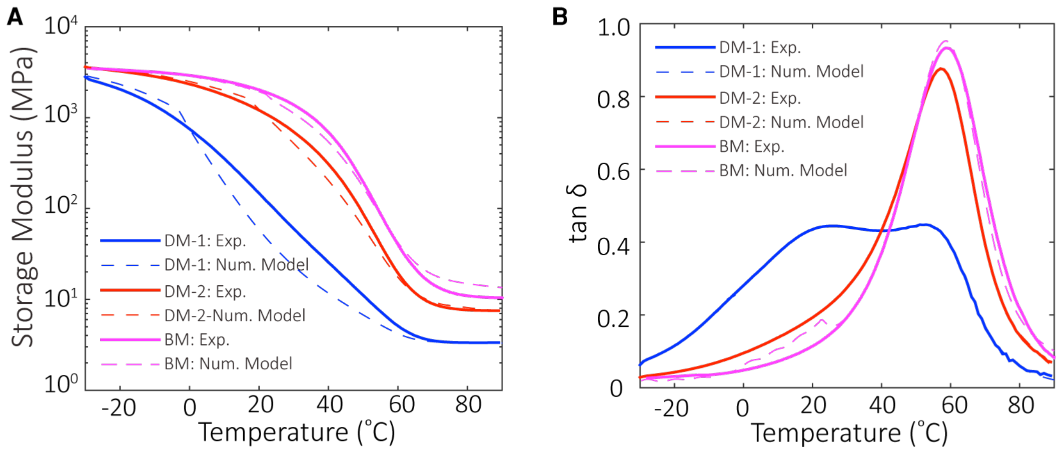

Figure S6 Comparison of the DMA curves between experimental data and numerical models for three SMP materials used in this paper.

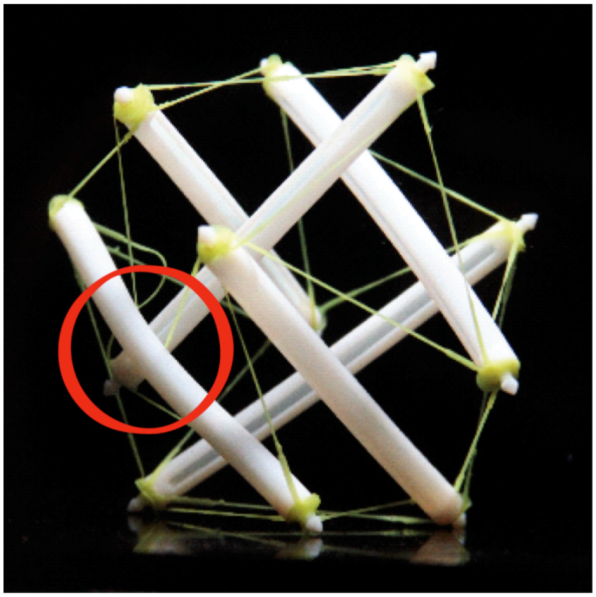

Figure S7 Failed deployment of a 6-strut spherical tensegrity, due to physical contact between struts, as highlighted by the red circle. As discussed in the main text, when the cables are loose, the folded struts are almost free to move in space. In this example, a strut blocks the recovery of another strut.

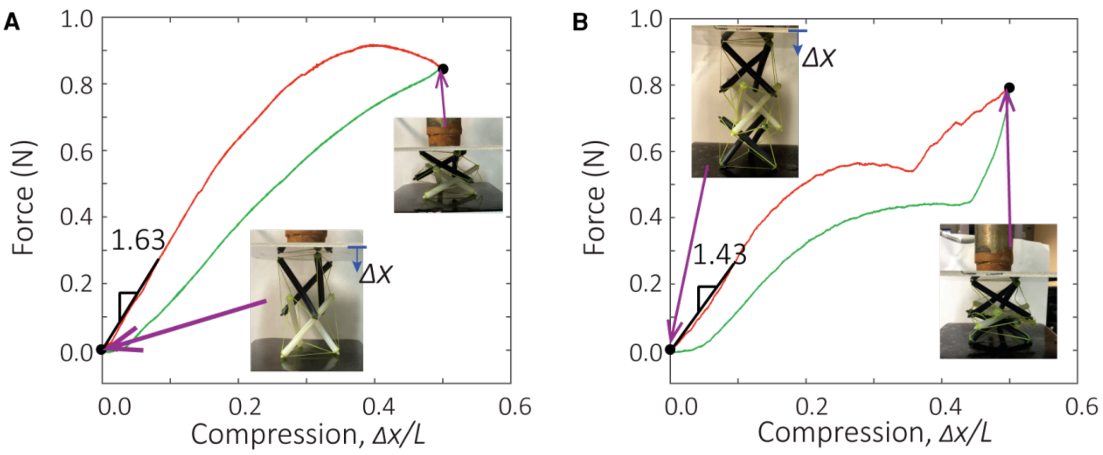

Figure S8 The compression tests on the two-layer tensegrity and the three-layer tensegrity, whose struts are made with two SMPs. (A) Compression test of the resultant deployed 2-layer tensegrity using 2 different SMPs for struts (BM and DM-1). The red line indicates the loading process while the green line indicates the unloading process. Estimated maximal compression equals 0.12N. (B) Compression test of the deployed 3-layer tensegrity made with 2 different SMPs (BM and DM-1). Maximal compression in the struts is estimated to be around 0.14N. The three-layer tensegrity shows two dips in both the loading and unloading process. This is due to the inherent multi-stability behavior of such structures. That is, the structure has a multiple local minima of stored energy at different configurations. For example, when one layer of the tower is fully flattened, the structure is at an alternative stable state (other than the fully deployed configuration). Due to the contact of struts, the other stable configurations cannot be reached. However, it still leads to a reduction in stiffness of the structure (snap through). The 3-layer tensegrity in B illustrates this effect more clearly than the one in Figure 4D of the main content because the structure in A has more DM-1 struts, which are less stiff than DM-2 and BM struts in room temperature (~25˚C). Thus, when a contact between struts happens, the DM-1 struts will bend, leading the structure slightly closer to the ideal alternative stable configuration, although this state cannot be fully reached.

Table S1.

Material parameters for the multi branch model.

	Verowhite		DM9895		DM8530	
Branch	E_{non} (MPa)	τ_i	E_{non} (MPa)	τ_i	E_{non} (MPa)	τ_i
E_1	148.7076	2.00E-08	300	0.0001	170	1E-07
E_2	119.7517	4.27E-07	275	0.000657	188	9.93E-07
E_3	131.9798	5.47E-06	296	0.003872	212	0.00001
E_4	147.1372	5.89E-05	305	0.02	239	9.08E-05
E_5	282.3444	0.000547	350	0.1	268	0.00074
E_6	320.9668	0.004524	378	0.576863	293	0.005374
E_7	354.2126	0.032439	292	3.401616	308	0.035368
E_8	427.2871	0.2	215	20	291	0.2
E_9	178.2132	1	147	96.82391	285	0.954957
E_{10}	143.8276	3.250259	95.213467	362.9461	138	3.182197
E_{11}	151.2221	9.451896	63.12765	1000	162	7.497457
E_{12}	162.8788	30.23741	62.0921	2671.527	178	25.11365
E_{13}	162.4149	100	52.099306	7912.87	153	87.11596
E_{14}	151.456	315.2367	42.374719	23498.79	133	283.7953
E_{15}	141.8913	927.9366	35.205449	71461.38	122	905.6253
E_{16}	111.7587	8849.219	27.897552	228551.6	112	3025.975
E_{17}	140.7818	2849.202	20.760769	726401	98.09554	10000
E_{18}	81.89721	25294.7	15.532429	2277776	83.26095	32677.22
E_{19}	52.68197	72900	11.281878	7091525	65.70456	96510.16
E_{20}	12.47854	653520.3	8.305791	21997171	59.12021	267333.4
E_{21}	28.03173	213000	5.959708	68236585	51.92218	773277.7
E_{22}	1.712558	5370000	4.351312	2.08E+08	44.76933	2339554
E_{23}	4.830405	2000000	3.329757	6.41E+08	34.59949	7613180
E_{24}	1.197657	85400000	2.644468	2.07E+09	21.72712	26070126
E_{25}	1.383214	20000000	2.196711	7.07E+09	9.995279	1E+08
E_{26}	0.000183	3.61E+08	1.578065	2.4E+10	2.916758	5.22E+08
E_{27}	2.537188	2E+09	0.1070122	1E+11	0.957138	5.77E+09
E_{eq} (MPa)	10.4		3.30		7.5	
T_g (˚C)	60		38		57	
T_{ref} (˚C)	22		-3		17	
C1	17.44		17.44		17.44	
C2	66.35		42.1		50.5	
AF_c/k	-23000		-23000		-24000	

Movie S1 Deployment of a 3-strut tensegrity.

Movie S2 Numerical simulation of the free recovery of a single strut with the slitted tubular cross section.

Movie S3 Deployment of a 6-strut spherical tensegrity, using the partial folding strategy.

Movie S4 Deployment of a 6-strut spherical tensegrity with 3 discrete attaching pieces of surface.

Movie S5 Deployment of a 6-strut spherical tensegrity with one continuous attaching piece of surface.

Movie S6 Programmed sequential deployment of a 2-layer tower tensegrity, whose struts are made with 2 SMPs.

Movie S7 Programmed sequential deployment of a 3-layer tower tensegrity, whose struts are made with 2 SMPs.

Movie S8 Programmed sequential deployment of a 3-layer tower tensegrity, whose struts are made with 3 SMPs.

References:

1 Guest, S. D. The stiffness of prestressed frameworks: A unifying approach. *International Journal of Solids and Structures* **43**, 842-854, doi:10.1016/j.ijsolstr.2005.03.008 (2006).

2 Zhang, J. & Ohsaki, M. *Tensegrity Structures - Form, Stability, and Symmetry*. (Springer Japan, 2015).

3 Calladine, C. R. The theory of thin shell structures 1888-1988. *ARCHIVE: Proceedings of the Institution of Mechanical Engineers, Part A: Power and Process Engineering 1983-1988 (vols 197-202)* **202**, 141-149, doi:10.1243/PIME_PROC_1988_202_020_02 (1988).

4 Timoshenko, S. P. & Gere, J. M. *Theory of Elastic Stability*. 2nd edn, (Dover Publications, 2009).

5 O'Connell, P. A. & McKenna, G. B. Arrhenius-type temperature dependence of the segmental relaxation below Tg. *The Journal of Chemical Physics* **110**, 11054, doi:10.1063/1.479046 (1999).

6 Williams, M. L., Landel, R. F. & Ferry, J. D. The Temperature Dependence of Relaxation Mechanisms in Amorphous Polymers and Other Glass-forming Liquids. *Journal of the American Chemical Society* **77**, 3701-3707, doi:10.1021/ja01619a008 (1955).

7 Di Marzio, E. A. & Yang, A. J. M. Configurational entropy approach to the kinetics of glasses. *J Res Natl Inst Stan* **102**, 135-157, doi:DOI 10.6028/jres.102.011 (1997).

8 Diani, J., Gilormini, P., Frédy, C. & Rousseau, I. Predicting thermal shape memory of crosslinked polymer networks from linear viscoelasticity. *International Journal of Solids and Structures* **49**, 793-799, doi:10.1016/j.ijsolstr.2011.11.019 (2012).

9 Sherrod, P. Nonlinear Regression Analysis Program, NLREG Version 5.0. *Phillip H. Sherrod, Nashville, TN* (2000).
